# The role of self-objectification and women’s blame, sympathy, and support for a rape victim

**DOI:** 10.1371/journal.pone.0199808

**Published:** 2018-06-28

**Authors:** Casey L. Bevens, Amy L. Brown, Steve Loughnan

**Affiliations:** 1 Department of Psychology, University of Edinburgh, Edinburgh, Scotland, United Kingdom; 2 Department of Psychology, University of Louisiana at Lafayette, Lafayette, Louisiana, United States of America; University of Westminster, UNITED KINGDOM

## Abstract

Sexual aggression is prevalent and damaging in our culture, and sources of support or blame following an attack of this kind can be important influences on the recovery process. This pair of studies investigate the nature of women’s blame reactions towards survivors of sexual aggression, as well as the potential for provision of sympathy and support. Specifically, we focused on the previously neglected role of female self-objectification. It was expected that increased self-objectification would lead to decreased sympathy and support, and more rape victim blame. However, results of Study 1 showed that chronic self-objectification was actually related to higher levels of sympathy and support for a rape victim. Study two built upon the limitations of study one, and examined similar questions. It was expected that women who engaged in greater self-objectification would again show greater sympathy and support for the victim, replicating study one’s results, and this was supported with a different scale. The overall relationship between self-objectification and sympathy and support was driven by body-relevant control beliefs. Implications and future directions are discussed.

## Introduction

Sexual aggression, which includes all acts of unwanted sexual contact, up to and including rape, is a major problem for women and girls [1, 2]. Additionally, college-aged women appear to be disproportionally victimized. Completed and attempted rape in this particular population was thoroughly measured via the National College Women Sexual Victimization Study (NCWSV) [3]. The NCWSV found an occurrence rate of 2.5% against women on U. S. college campuses over a six-month period. While this rate may seem small, at a University with an enrollment of 20,000 students, this translates to the potential for up to 250 women being assaulted during a single school year. Stated another way, over the seven-month period of a school year, this would mean more than one rape or attempted rape occurring per day, per campus. While students represent a population that is particularly affected by sexual aggression, a discussion of these types of crimes is incomplete without consideration of the wider group of all women and girls.

In a work that focused on college women, but also included the research surrounding sexual aggression against all women [4], authors thoroughly discussed the methodology, results, and implications surrounding rape and sexual aggression statistics [5–8]. Results of this cluster of research initiatives indicate collectively a systematically prevalent burden on female students, which is not experienced similarly by male students on college campuses, regarding maintaining personal safety specific to sexual aggression. Additionally, these studies can be seen as contributing to evidence of a larger pattern of aggression against women and girls, where the gender group as a whole is targeted, primarily by men, for sexual crimes [9–13]. Taking into account both the demonstrable subjection of women in general to the threat of sexual aggression, as well as the more focused targeting of the sub-set of college women for victimization, this paper will focus on two samples. We collect data from both a college sample of women (Study 1) and a broader online sample of women (Study 2).

When incidents of rape occur, women are highly unlikely to report to the police; only about 2% of victims do so [14, 15]. Although women rarely report to the police, often out of fear of being blamed or not believed, survivors of sexual aggression are sharing with other people in their lives. Among victims who choose to share their experience with someone other than the police, the majority of the time the person chosen as a confidante will be a friend [14, 15]. Although the reasoning behind turning to a friend following such a trauma is often to elicit emotional support [14, 16], survivors are met with negative reactions as frequently as 39% of the time. These reactions may include victim blame and rejection [14, 17]. Whereas positive reactions have little to no effect on recovery from the trauma, negative reactions ultimately are very harmful to the psychological wellbeing of survivors [18], and blaming reactions from those sought out for support have been found to be related to higher rates of re-victimization over a twelve-month period [19]. In short, the responses of friends matter deeply.

We examined the role of self-objectification in women’s reactions to female sexual aggression victims. Objectification Theory [20] asserts that women are socialized to view themselves as objects to be evaluated based on appearance. Three major points make up the process of self-objectification in the Fredrickson and Roberts model: the internalization of appearance ideals, valuing appearance over competence, and body surveillance. Often, this is accomplished through media, which plays a major role in the sexual socialization of young people [21, 22]. When objectification is involved in media ideals, and those ideals serve to target the male gaze, there may be an implied message that men have the right to both look and touch, because objects lack autonomy in their own right. Put another way, rape can be seen as “making good on the threat of sexual objectification” [23].

It is not illogical to think that those who are particularly focused on their own bodily presentation would also tend to scrutinize the appearances of others. Indeed, women who have a strong focus on their own bodies and shape have a tendency to focus on the shape of other women and also tend to assume that those other women will have a similarly strong emotional investment in their own bodies [24]. When women who self-objectify then go on to objectify other women, a “circle of objectification” [25] can be said to exist. If women objectify other women in everyday life, they may also do so cases of sexual victimization, especially as this type of crime involves treating another person as a body.

Objectification of victims of sexual aggression does play a role in perceptions of those victims and their responsibility for having been victimized. Both men and women tend to objectify sexualized women via a withdrawal of moral concern and mind [26]. When that sexualized woman has been the victim of rape, this additionally results in her being attributed greater responsibility for the assault. This work also found evidence for a tacit, indirect denial of suffering in the case of the sexualized woman, as indicated in the lowered moral concern for her well-being. Thus, objectification extends beyond everyday interactions and plays a role in sexual aggression.

In addition to the theorized link with self-objectification, differences in perception of victims may be influenced by individual factors such as the extent to which a given women endorses rape myths. It has been suggested that a major logical inference of rape myth acceptance is less sympathy for victims [27], and given that most rape victims will turn to a friend, often a female friend, for support, there are clear negative implications for the impact of rape myth acceptance by women on the likelihood that support will be given when needed. The employment of rape myths indeed has been shown to play a role in how women define and interpret scenarios that meet the legal definition of rape, but which include elements of myths and do not fall into the socially proscribed ideas found in the common rape script [28]. Thus, measures of rape myth acceptance were included in both studies presented here.

In instances of sexual aggression, this way of thinking may also be reflected in higher rates of victim blaming for occurrences of acquaintance rape vs. stranger rape [29–31], because it is easier to blame a victim who knew her attacker than one whose rape was consistent with the “stranger in the bushes” stereotype. When characteristics exist in a rape scenario that could be viewed as foreseeably contributing to the assault, such as previous acquaintance between victim and perpetrator, or victim’s use of alcohol or drugs, it may be easier for observers to assume that she “should have known” enough to prevent the attack. In such cases where the victim’s behavior can be seen as a potential cause of the assault, it may be easier for people to attribute blame to the victim (upholding the belief in a just world where bad things only happen to those who deserve them), than in cases where victim-related causal factors are harder to identify [32]. These tendencies are reflected in the choice of victims and types of scenarios used in the present studies.

### The present studies

It is the purpose of the current set of studies to investigate the nature of victim blaming engaged in by women, directed at survivors of sexual aggression, as well as the potential for provision of sympathy and support to victims by other women in relation to their own self-objectification. While much of the research suggests a stronger effect of victim blame for men than for women [31], it is particularly relevant to examine the interactions that exist between rape victims and other females, as these may be a source of much of the social support from which women victims draw. Victims may want to turn to other women for support and understanding, but find that these are not forthcoming, or that they are met with ambivalent attitudes. It is possible that self-objectification plays a role in exacerbating these types of problematic responses. By focusing on a women-only group for this study, it is hoped that we will further the understanding of the processes that contribute to the challenges victims of sexual aggression may face following the event, which include possible re-victimization by way of rejection and lack of understanding from social groups.

## Study one

Study one investigated victim blaming and sympathy and support towards victims of sexual aggression. Study One was conducted in the context of a larger study, which attempted to elicit state self-objectification via exposure the objectifying media. Details of this broader study can be found in the online supplementary materials (https://osf.io/2u7z6/). The primary intention of the study reported here was to examine whether self-objectification would inhibit sympathy and support for victims of sexual aggression, and effect greater attributions of victim responsibility for being raped in a sample of college women. It was further proposed that rape myth acceptance may act as a moderator of these relationships. Specifically, we hypothesized the following:

H1. Participants who have higher levels of overall trait self-objectification will have lower levels of sympathy and support for a victim of rape and higher levels of victim blame.
H2. The relationship between self-objectification and sympathy and support will be moderated by participants’ pre-existing rape myth acceptance, such that the hypothesized negative relationship between self-objectification and sympathy and support will strengthen as rape myth acceptance increases.
H3. The relationship between self-objectification and victim blame will be moderated by participants’ pre-existing rape myth acceptance, such that the hypothesized positive relationship between self-objectification and victim blame will strengthen as rape myth acceptance increases.

### Study one methods

#### Participants

A power analysis indicated a need for 168 participants to detect a medium effect size with 80 percent power. A total of 207 women participated over the course of 20 sessions, which were held at the end of the fall 2015 and the beginning of spring 2016. The participants were undergraduate females over the age of 18 recruited from the participant pool system at a university in the American south for course credit. Eight participants were excluded from analyses for getting less than three out of four minimum attention check questions correct following the viewing of the film clip. Ten participants were excluded from final analyses for choosing not to respond to at least two dependent variable items. A final sample of 189 participants remained that were included in analyses (mean age = 18.88 years). Of the women sampled, 113 identified as White, 55 as African America, 6 as Hispanic or Latino, 6 as Asian, 5 as Other, and 4 did not provide this information. This study was approved by the Institutional Review Board of the University of Louisiana at Lafayette, and written participant consent was obtained individually at the time of each study session.

#### Materials

All materials and measures can be viewed in the online supplementary materials (https://osf.io/2u7z6/). The Illinois Rape Myth Acceptance Scale- Updated version (IRAMA-U) [33] was used to measure participants’ rape myth acceptance. The scale is an updated version of the original Illinois Rape Myth Acceptance Scale [34], and attempts to account for changes in the way that people use language to describe rape that may have occurred since the creation of the IRMA, as well as subtleties in descriptions of rape myths. This scale includes 22 items (1, strongly disagree; 5, strongly agree) that measure five factors: it wasn’t really rape, he didn’t mean to, he didn’t mean to/ intoxication related items, she lied, and she asked for it. Cronbach’s alpha for the scale was 0.90 in this sample, and a single mean score was created across all factors for each participant.

The Self-Objectification Questionnaire (SOQ) [35] was used to measure participant self-objectification. This scale includes five body attributes that are appearance-based, and five that are competence based, and participants are asked to rank each of the ten areas from 1 (least important to them) to 10 (most important to them). Scores are determined by separately summing the appearance and competence scores, then subtracting the sum of the competence ranks from the sum of the appearance ranks, and range from -25 to 25. Higher scores reflect a greater emphasis on personal appearance in forming a body-relevant self-concept, which is interpreted as high trait self-objectification.

A scene taken from the 1988 film *The Accused* was used as the depiction of rape for this study, lasting four minutes and 28 seconds. The video can be found online at https://www.youtube.com/watch?v=qrBeQnqZXu0&t=1s. This depiction of sexual aggression contains elements of several common rape myths, but is not a representation of what would be deemed a pure date rape scenario. The scene depicts a young woman in the back room of a bar drinking alcohol, smoking marijuana, and flirting with a man she met that night. The man begins to attempt to engage her sexually and she says that she needs to work in the morning and pushes him away. Despite her verbal protests and attempts to physically stop it, he then rapes her on top of a pinball machine while many other men look on. The original scene depicts several more men raping the victim, but for the purposes of this study the scene was only shown up to the point of the beginning of the first rape. Rather than showing the rape, the clip was ended, and participants were informed that a rape then occurred. This decision was made with participant interests in mind, due to the fact that the lengthier version may have been more traumatic to viewers. There were also practical and theoretical considerations, in that the full scene is rather long, and that showing a depiction of rape or gang rape may affect participant reactions differently from a simple factual description of a single rape. Written vignettes, common to this type of research, are not graphic in their descriptions of the act of rape, while a visual depiction is by nature more graphic. Thus, the act itself was not shown. The film was chosen in part because this study deals with media in general, so a media representation, as opposed to written vignettes, seemed appropriate. The film is also old enough that it is less probable that many participants would have seen it before. Indeed, only four women indicated that they were familiar with the film from which the clip was taken.

In order to assess sympathy and support for the victim of rape, six questions were written (e.g. How willing would you be to provide emotional support to Sarah; 1, not at all willing; 9, very willing). Higher scores indicate greater sympathy and support with the woman depicted in the scene, while lower scores indicate less sympathy and support for the woman for this scale (α = 0.85). To examine Victim Blame, five questions were created (e.g. How much do you believe that what happened was Sarah’s fault?; 1, not at all; 9, very much). Questions about the woman’s behavior indicate greater blame when higher scores were endorsed for this scale (α = 0.83). A final set of five questions were exploratory in nature, and included such items as “How realistic do you find this scene?” as well as questions assessing perceived intoxication of the characters.

#### Procedure

Participation took place in a single session of approximately thirty minutes in length that was overseen by the first author. Materials were presented via a web-based survey conducted in person in a computer lab at the University. Brief demographic information was taken, and participants viewed and responded to an initial set of questionnaires that included the Multidimensional Body-Self Relations Questionnaire [36], measuring body dissatisfaction; the Eating Attitudes Test-26 [37], measuring eating pathology; and the Illinois Rape Myth Acceptance Scale- Updated (IRMA-U) [33], measuring rape myth acceptance. Of these, only the IRMA-U was used in these analyses, and thus it is the only one with reported results. Next, based on random assignment, as part of the larger study not reported in detail here, participants viewed the set of print media images in order to attempt to manipulate self-objectification. Following this, participants filled out the Self-Objectification Questionnaire [35]. At this point, the subjects were shown the rape scene from *The Accused*. Finally, they were asked to fill out the questionnaire measuring the outcome variables of sympathy and support for the depicted victim and participant attribution of blame to the victim, along with the exploratory questions and items serving to check that they attended to the film. Participants all received a debriefing form which explained the purpose of the study, and the researcher was on hand to answer any questions and take note of any comment that arose.

### Results

All underlying data for the following analyses are fully available in the online supplementary materials (https://osf.io/872qs/). Prior to testing the hypotheses, a preliminary examination of all the study variables’ means was conducted. They were all found to have a reasonably normal distribution, and correlations and descriptive statistics for the main variables can be found in Table 1.

**Table 1:** Correlations and descriptive statistics among study one variables.

	*SOQ*	*IRMA-U*	*Sympathy/Support*	*Victim Blame*
Variable				
SOQ	1			
IRMA-U	-.007	1		
Sympathy/Support	.140	-.336**	1	
Victim Blame	-.128	.599**	-.544**	1
Descriptive Statistics				
Mean	0.12	2.11	5.37	3.75
Standard Deviation	13.25	0.62	1.75	1.90

Note.

*p < 0.05, two-tailed

**p < 0.01, two tailed.

#### Hypothesis 1

To test hypothesis one, that participants who had higher levels of self-objectification would have lower levels of sympathy and support for a victim of rape and higher levels of victim blame, simple regression was employed. Self-objectification was expected to predict low sympathy and support in one model, and high victim blame in the second model.

This hypothesis was not supported for victim blame regressed on self-objectification, *b* = -0.02, *S*.*E*. = 0.01, *t* = -1.76, *p* = 0.08. However, for the relationship between sympathy and support and self-objectification, there was a significant effect, although this was not in the predicted direction, *b* = 0.02, *S*.*E*. = 0.01, *t* = 1.93, *p* = 0.05. So, women who engaged in higher overall self-objectification showed more sympathy and support for the victim.

#### Hypothesis 2

Hypothesis two predicted that the relationship between self-objectification and sympathy and support would be moderated by participants’ rape myth acceptance. We tested this using the PROCESS macro for SPSS, and found that there were main effects of self-objectification (*b* = .02, *S*.*E*. = .01, *t* = 2.11, *p* = .04) and rape myth acceptance (*b* = -.99, *S*.*E*. = .20, *t* = -4.81, *p*< .001) on sympathy and support. However, the interaction term of self-objectification and rape myth acceptance was non-significant (*b* = .02, *S*.*E*. = .02, *t* = 1.21, *p* = .23), indicating that moderation via rape myth acceptance was not supported.

#### Hypothesis 3

Our third hypothesis expected that the relationship between self-objectification and victim blame would be moderated by participants’ rape myth acceptance, and was also tested using the PROCESS macro for SPSS. There were significant main effects of self-objectification (*b* = -.02, *S*.*E*. = .01, *t* = -2.20, *p* = .03) and rape myth acceptance (*b* = 1.89, *S*.*E*. = .11, *t* = 9.34, *p*< .001). However, their interaction was non-significant (*b* = -.03, *S*.*E*. = .02, *t* = -1.81, *p* = .07), indicating that moderation was not supported.

#### Exploratory analyses

Simple regression was employed to assess whether the perceived level of victim intoxication influenced sympathy and support or victim blame. There was no relationship between victim blame and perceived victim intoxication, *b* = 0.15, *S*.*E*. = 0.09, *t* = 1.68, *p* = 0.10. There was a negative relationship between sympathy and support and intoxication for the victim, *b* = -0.16, *S*.*E*. = 0.08, *t* = -1.94, *p* = 0.05. This is not surprising, considering that intoxication is an element of common rape myths and that the clip was chosen specifically with rape myths in mind. Simple regression was also used to assess whether the perceived level of perpetrator intoxication influenced either sympathy and support or victim blame. However, there was no relationship between how intoxicated participants perceived the perpetrator to be and sympathy and support for the victim, *b* = -0.05, *S*.*E*. = 0.06, *t* = -0.87, *p* = 0.39, or between perceived perpetrator intoxication and victim blame, *b* = 0.07, *S*.*E*. = 0.06, *t* = 1.16, *p* = 0.25.

## Study two

Study one garnered surprising results that did not confirm our original hypotheses. In particular, we found a positive relationship between self-objectification and sympathy and support. In light of this, we sought to produce a replication using stronger methodology, in order to better determine whether this represents a true effect prior to offering any firm interpretations thereof. In study two, we decided to use a vignette of a rape disclosure that was pilot tested for realism, in order to be more relevant to the elicitation of reactions to disclosures of rape. In addition, this study used alternative, well validated, scales to measure self-objectification, sympathy and support, victim blame, and rape myth acceptance. The following hypotheses were tested based on the literature and the results of study one:

H1. Participants who have higher levels of overall self-objectification will have higher levels of sympathy and support and higher levels victim blame for a victim of rape, consistent with ambivalent attitudes.
H2. The relationship between self-objectification and sympathy and support will be moderated by participants’ pre-existing rape myth acceptance, such that the positive relationship between self-objectification and sympathy and support will weaken as rape myth acceptance increases.
H3. The relationship between self-objectification and victim blame will be moderated by participants’ pre-existing rape myth acceptance, such that the positive relationship between self-objectification and victim blame will strengthen as rape myth acceptance increases.

### Methods

#### Participants

A sample of 105 women over the age of 18 who identified as British nationals participated in study two. They were recruited online, and received £1 for their time. No other demographic information was collected, in consideration of the sensitive nature of the subject matter. Of these, two did not complete more than 20% of the materials and were excluded, leaving a final sample of 103. This study was approved by the University of Edinburgh ethics committee, and consent was obtained by participants’ choice to click to continue into the study following reading an information sheet online.

#### Materials

All materials and measures can be found in the online supplementary materials (https://osf.io/2u7z6/). In order to measure self-objectification of the participants, the SOQ [35] was used again for replication purposes. In addition, the construct was measured using the Objectified Body Consciousness Scale (OBCS) [38]. This 24-item scale (α = .86; measured from 1, strongly disagree- 7, strongly agree) consists of three factors: Body Shame (e.g. “I feel ashamed of myself when I haven’t made the effort to look my best;” α = .88), Body Relevant Control Beliefs (e.g. “I think a person can look pretty much how they want to if they are willing to work at it;” α = .77), and Body Surveillance (e.g. “During the day I think about how I look many time;” α = .87).

The rape vignette employed here was based on a vignette used in past research [39, 26] and was altered to reflect a first-person disclosure by an acquaintance named Laura. The vignette was also pilot tested in this study for perceived realism, after which minor alterations to wording were made.

Sympathy and support for the victim was measured using a six-item scale (α = .85; possible range 1–5) [39]. An example item from the sympathy and support scale is “How much sympathy do you have for Laura.” Victim blame was assessed using a six-item scale (α = .84) taken from these same authors, which included items such as “To what extent was Laura’s behavior responsible for her sexual encounter with the man.”

Rape myth acceptance was measured with the twenty-five-item Attitudes Towards Rape Victims Scale (ARVS) [40]. An example item from this scale is “The extent of the woman’s resistance should be the major factor in determining if a rape has occurred,” measured from 1(strongly disagree) to 5 (strongly agree), α = .85. Objectification of the victim was also measured, using a modified SOQ, but this was perfectly correlated with the SOQ, and thus is not reported any further.

#### Procedure

Participation took place online and was anonymous. Following informed consent, participants were first presented with the SOQ and OBCS. They then read the rape vignette, which was followed by the measures of sympathy and support and victim blame, the modified SOQ, and the ARVS in that order. Following this, they were directed to a debriefing page and the study concluded.

### Results

All underlying data for the following analyses are fully accessisble in the online supplementary materials (https://osf.io/4fjy7/). Prior to testing the hypotheses, a preliminary examination of all the study variables’ means was conducted to determine normality and test for skewness. They were all found to have a reasonably normal distribution, with exception of the positively skewed variables of Victim Blame and the ARVS, a pattern which is not altogether unsurprising. Correlations and descriptive statistics for all study 2 variables can be found in Table 2.

**Table 2:** Correlations and descriptive statistics among study two variables.

	SOQ	OBCS Overall	OBCS Body Surveillance	OBCS Body Shame	OBCS Control Beliefs	Victim Blame	Sympathy/ Support	ARVS
Variable								
SOQ	1							
OBCS Overall	-.143	1						
OBCS Body Surveillance	-.230*	.842**	1					
OBCS Body Shame	-.173	.831**	.624**	1				
OBCS Control Beliefs	.180	.322**	-.012	-.082	1			
Victim Blame	-.035	-.061	-.076	.004	-.066	1		
Sympathy/Support	.074	.206*	.131	.077	.262**	-.226*	1	
ARVS	-.054	-.110	-.040	-.003	-.237*	.662**	-.348**	1
Descriptive Statistics								
Mean	0.44	4.18	4.36	3.67	4.50	1.36	4.40	1.48
S.D.	14.63	0.79	1.19	1.29	0.88	0.53	0.55	0.44

Note.

*p < 0.05, two-tailed.

**p < 0.01, two tailed.

#### Hypothesis 1

Hypothesis 1 tested whether participants with higher levels of overall self-objectification would have higher levels of sympathy and support and higher levels of blame for a victim of rape. When the SOQ was regressed on sympathy and support, no significant relationship was found (*b* = .003, *S*.*E*. = .004, *t* = .738, *p* = .462), thus failing to replicate our findings from Study 1 with this scale. There was also no significant relationship found between the SOQ and Victim Blame (*b* = -.001, *S*.*E*. = .004, *t* = -.349, *p* = .728).

However, when self-objectification was measured using the Overall OBCS, including all three subscales, there was a significant relationship between self-objectification and sympathy and support (*b* = .143, *S*.*E*. = .068, *t* = 2.116, *p* = .037). When broken down by sub-scales, there was no significant relationship between Body-Surveillance and Sympathy and Support (*b* = .060, *S*.*E*. = .045, *t* = 1.332, *p* = .186), or between Body Shame and Sympathy and Support (*b* = .033, *S*.*E*. = .042, *t* = .776, *p* = .440). There was a significant relationship between the Control Beliefs sub-scale and Sympathy and Support (*b* = .163, *S*.*E*. = .060, *t* = 2.725, *p* = .008). For the outcome variable of Victim Blame, there was no significant relationship with the Overall OBCS (*b* = -.042, *S*.*E*. = .069, *t* = -.608, *p* = .544), the Body Surveillance sub-scale (*b* = -.034, *S*.*E*. = .045, *t* = -.754, *p* = .452), Body Shame sub-scale (*b* = .002, *S*.*E*. = .041, *t* = .040, *p* = .968), or the Control Beliefs sub-scale (*b* = -.041, *S*.*E*. = .062, *t* = -.662, *p* = .509).

#### Hypothesis 2

Hypothesis 2 tested whether the relationship between self-objectification and sympathy and support would be moderated by rape myth acceptance, using the PROCESS macro for SPSS. This hypothesis was not supported for any of the measures of self-objectification tested. When self-objectification was measured with the SOQ, there was a main effect of the ARVS (*b* = -.457, *S*.*E*. = .151, *t* = -3.026, *p* = .003), but no effect of self-objectification (*b* = .002, *S*.*E*. = .004, *t* = .506, *p* = .614), or their interaction term (*b* = -.010, *S*.*E*. = .010, *t* = -.943, *p* = .348), indicating a lack of moderation. For the OBCS Overall, there was also a main effect of the ARVS (*b* = -.426, *S*.*E*. = .154, *t* = -2.758, *p* = .007), but no significant effect of self-objectification (*b* = .113, *S*.*E*. = .065, *t* = 1.752, *p* = .083), or the interaction term (*b* = -.089, *S*.*E*. = .178, *t* = -.503, *p* = .616). For the OBCS Body Surveillance scale, there was a main effect of the ARVS (*b* = -.463, *S*.*E*. = .156, *t* = -2.978, *p* = .004), no main effect of body surveillance (*b* = .056, *S*.*E*. = .047, *t* = 1.178, *p* = .243), and no significant effect of the interaction (*b* = -.135, *S*.*E*. = .124, *t* = -1.089, *p* = .279). The same pattern emerged for the Body Shame scale, with a main effect found for the ARVS (*b* = -.436, *S*.*E*. = .135, *t* = -3.238, *p* = .002), and no effect of either Body Shame (*b* = .033, *S*.*E*. = .044, *t* = .734, *p* = .465), or the interaction term (*b* = .091, *S*.*E*. = .119, *t* = .764, *p* = .447). Lastly, for the Control Beliefs scale, there were main effects for both the ARVS (*b* = -.412, *S*.*E*. = .145, *t* = -2.836, *p* = .006) and Control Beliefs (*b* = .118, *S*.*E*. = .053, *t* = 2.215, *p* = .029), but the interaction of the two was non-significant (*b* = -.135, *S*.*E*. = .153, *t* = -.886, *p* = .378).

#### Hypothesis 3

**Fig 1:**
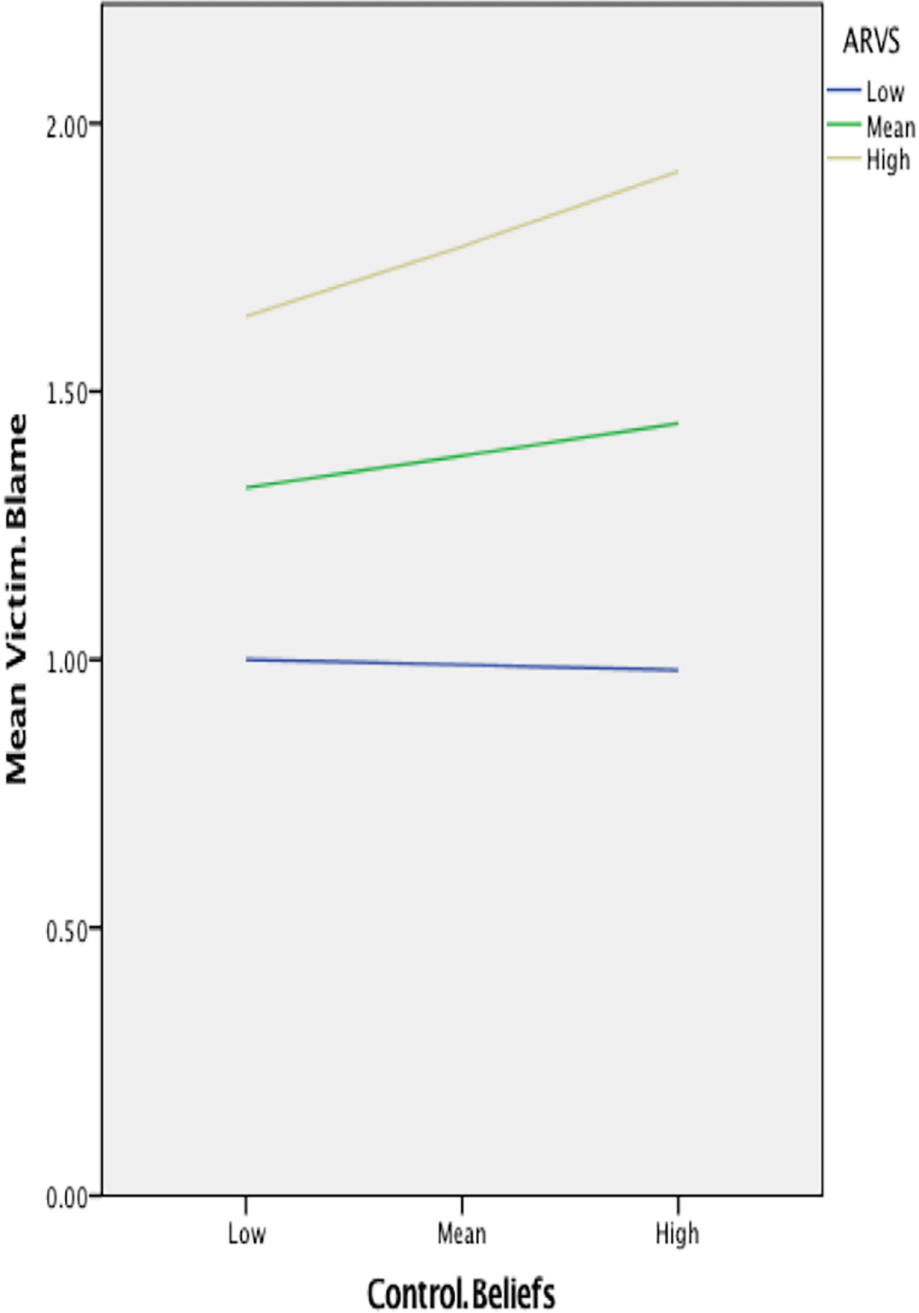
Simple slopes equations of the regression of victim blame on control beliefs at three levels of rape myth acceptance.

Hypothesis 3 examined whether the relationship between self-objectification and victim blame would be moderated by rape myth acceptance, also using the PROCESS macro for SPSS. This hypothesis was supported for the OBCS Control Beliefs scale, where there was a significant effect of the ARVS, *b* = .894, *S*.*E*. = .174, *t* = 5.137, *p*< .001; a non-significant effect of Control Beliefs, *b* = .071, *S*.*E*. = .040, *t* = 1.792, *p* = .076; and a significant interaction of the two, *b* = .194, *S*.*E*. = .083, *t* = 2.332, *p* = .023. Simple slopes analyses revealed that when rape myth acceptance (ARVS) is high, there is a significant effect of Control Beliefs on Victim Blame, *b* = .157, *S*.*E*. = .064, *t* = 2.434, *p* = .017; but when rape myth acceptance is average (*b* = .071, *S*.*E*. = .040, *t* = 1.792, *p* = .076), or low (*b* = -.014, *S*.*E*. = .041, *t* = -.342, *p* = .733), this effect becomes non-significant (see Fig 1). Continuous moderation was run, but these data are visualized at the mean of the moderator (ARVS) and ±1 standard deviation of the mean of the moderator.

There was no evidence of moderation in the case of the other measures of self-objectification. When self-objectification was measured with the SOQ, there was a main effect of the ARVS (*b* = .812, *S*.*E*. = .173, *t* = 4.610, *p*< .001), but no effect of self-objectification (*b* = -.000, *S*.*E*. = .003, *t* = -.081, *p* = .936), or their interaction term (*b* = -.000, *S*.*E*. = .012, *t* = -.020, *p* = .984), indicating a lack of moderation. For the OBCS Overall, there was also a main effect of the ARVS (*b* = .849, *S*.*E*. = .182, *t* = 4.653, *p*< .001), but no significant effect of self-objectification (*b* = .026, *S*.*E*. = .060, *t* = .430, *p* = .668), or the interaction term (*b* = .220, *S*.*E*. = .188, *t* = 1.172, *p* = .244). For the OBCS Body Surveillance scale, there was a main effect of the ARVS (*b* = .785, *S*.*E*. = .168, *t* = 4.679, *p*< .001), no main effect of body surveillance (*b* = -.019, *S*.*E*. = .040, *t* = -.470, *p* = .640), and no significant effect of the interaction (*b* = -.059, *S*.*E*. = .134, *t* = -.436, *p* = .664). Lastly, the same pattern emerged for the Body Shame scale, with a main effect found for the ARVS (*b* = .801, *S*.*E*. = .155, *t* = 5.181, *p*< .001), and no effect of either Body Shame (*b* = .003, *S*.*E*. = .034, *t* = 1.312, *p* = .927), or the interaction term (*b* = .175, *S*.*E*. = .134, *t* = 1.312, *p* = .193).

## General discussion

In two studies, we investigated whether women’s self-objectification effects women’s perceptions of rape victims, and whether rape myth acceptance might moderate this relationship. We found that self-objectification was related to sympathy and support, such that greater self-objectification predicated higher sympathy and support for the victim of rape. When this was broken down, the effect appeared to be driven by body-relevant Control Beliefs. Furthermore, rape myth acceptance moderated the relationship between Control Beliefs and Victim Blame.

These results indicate that higher self-objectification is linked to greater sympathy and support, perhaps via objectified women seeing themselves as lesser than others. This may create a hyper-humanizing perception of victims. This proposed effect is consistent with research indicating that women with eating disorders, who can be conceptualized as engaging in extreme forms of self-objectification, often tend to suppress negative emotions, particularly anger [41], and operate in a caretaking role for others [42, 43]. This type of heightened interpersonal orientation may play a role in objectified women forming a sense of cohesion with a victim of sexual aggression.

It is also possible that women who self-objectify to a greater degree are likely to relate more readily to a victim. Consistently experiencing the self as a body may make them better able to relate to the idea of being assaulted as a body. Furthermore, the results of study one are consistent with past literature which has shown that women may engage in a reactive way, or show a “boomerang effect” [22] to objectifying imagery and media. In such studies, women have been shown to exhibit less tolerance of violent attitudes towards women in general following exposure to media of this kind [44–46]. It is also possible that highly objectified women are simply more in touch with the experience of womanhood in general, seeing this as more central to their self-concept than other women. As womanhood is often intertwined with the experience of objectification, they may be more likely to display a disposition of relating well to their in-group of women who are treated in similarly objectifying ways.

The exact means that self-objectification operates in this context, however, remains somewhat unclear based on our results. This is a multi-faceted theoretical construct, which we attempted to address to a certain degree in Study 2 through the use of additional measures. Due to our lack of replication for the SOQ, interpreting the role of that particular operationalization of self-objectification must be undertaken with some caution. Turning to the measurement of self-objectification with the OBCS, the role of the control beliefs as a driving force for sympathy and support is an interesting result. It is possible that women who are especially invested in controlling their own bodies see a violation of this control, which sexual aggression represents an extreme form of, as even more heinous. They therefore may relate more to female victims and exhibit the sympathy and support that was evident in our results.

Of additional interest is the moderating effect we found for Control Beliefs and Victim Blame by rape myth acceptance. Women who had higher levels of rape myth acceptance showed a relationship between Control beliefs and exhibiting Victim blame. This effect was consistent with predictions, and could have implications for understanding who is best equipped to be sought for support following events of sexual aggression. It cannot be assumed that simply because a potential confidante is also a woman that she will be empathetic towards a disclosure of sexual assault and not engage in victim blaming. Although to a lesser degree than men, women do endorse rape myths, and this does influence reactions to victims [47, 48]. Indeed, the results related to sympathy and support combined with these concerning victim blame are more consistent with an overall pattern of ambivalence towards victims among women (e.g. [49]).

While it would be nice to take away the simple idea that aftercare could use these results to inform survivors of clear strategies for carefully choosing confidantes that would take into account personally known characteristics of their peers, friends, family members, and other sources of social support, it is not that straightforward. When a possible female confidante is being considered, the ambivalence of our results represents a high-risk, high-reward scenario. On one hand, women who are higher in self-objectifying and Control Beliefs about themselves are more likely to exhibit more sympathy and support. However, women who similarly endorse high Control Beliefs but also endorse rape myths are more likely to engage in victim blame. A survivor should not simply seek women who show tendencies towards higher self-objectification, but ones they know well enough to trust do not endorse rape myths.

### Limitations and future directions

There are several limitations that should be considered when interpreting the results of this set of studies. In Study One we used a film rather than a written vignette. Although this has been done in other research on sexual aggression [29, 30, 50, 51], it may have represented a confounding variable. It was hoped that the use of the film would be more realistic to participants, and indeed they did rate the clip as realistic. However, this realism may have come at the price of experimental control. The use of vignettes, consistent with past literature on sexual aggression may be preferable, even in the case of studies concerning media perceptions. In Study Two the use of a first-person disclosure vignette, in addition to being better controlled, also represented a closer approximation of a real-world disclosure experience.

A second limitation that should be addressed is the fact that, while past research that informed this work indicates that rape victims are likely to disclose to friends [14, 15], our studies focused on a non-specified relationship between the participants and the victim (study 1) and a relationship with a victim described as an acquaintance (study 2). It is possible that reactions to disclosures by a person who is less well known could diverge from those reported by a close friend. However, we believe it is probable that disclosures are more likely to occur to other women (vs. men) in general. This work thus expands our knowledge by widening the scope of possible disclosure recipients from those of close friends to include the a wider range of other women. Other women beyond close friends may be especially relevant within the college sample used in study 1. Arguably, this sample represents a group of women who may have changed peer-groups relatively recently following high school, and will thus have less close long-term friends who are readily available. Thus, they may have to fall back on disclosing to other women within a wider peer group. Reactions from other women in general therefore are equally important to understand.

Despite being the most popular measure in the literature, the SOQ only taps one element of the theoretically multi-faceted [20] experience of self-objectification. We attempted to remedy this in study two, and had some success, despite the lack of replication of our results concerning the SOQ. Future research in this field should aim to determine when and where certain measures, which have generally been used interchangeably, are more appropriate. It should also seek to develop more concept specific measures of other aspects of objectification.

Our most important finding, that self-objectification predicted greater sympathy and support for a victim, merits further research. It is possible that the collective experience by women of objectification could be a point of cohesion within female groups that, although generally negative, is something that can be related to following commonly experienced instances of sexual aggression.

## Conclusions

This set of studies sought to expand on the growing body of literature concerning the role of objectification in perceptions of rape victims. Theoretical perspectives, drawn from Objectification Theory, as well as literature on sexual aggression informed the present study. We examined the role that self-objectification among women has on sympathy, support, and victim blame in a rape, as well as the possibility that the individual differences characteristic of rape myth acceptance may affect this relationship.

The role of objectification in perceptions of victims of rape remains an important area for consideration in further research. The present study aimed to further understand that relationship. We also sought to fulfil an important recommendation by Moradi and Huang [52] that more research should examine women’s safety anxiety, specifically anxiety about sexual aggression that may accompany experiences of objectification. These results also raise questions for future research into ways that women can best serve as sources of support to other women following incidences of sexual aggression, and how support may be best delivered and defined. We observed a novel and surprising relationship between women’s self-objectification and *heightened* sympathy and support for a rape victim. This adds to our understanding of women’s interactions with one another and potential to provide support following violent acts that disproportionately affect the gender as a whole.
